# Computational study on organochlorine insecticides extraction using ionic liquids

**DOI:** 10.1016/j.heliyon.2024.e25931

**Published:** 2024-02-12

**Authors:** Mohammad K. Al Hassan, Mustafa S. Nasser, Ibnelwaleed A. Hussein, Muneer Ba-Abbad, Imran Khan

**Affiliations:** a Gas Processing Center, College of Engineering, P.O. Box 2713, Qatar University, Doha, Qatar; b Chemical Engineering Department, College of Engineering, P.O. Box 2713, Qatar University, Doha, Qatar; c Department of Chemistry, College of Science, Sultan Qaboos University, Muscat, Oman

**Keywords:** Ionic liquids, Computational study, Organochlorine, Insecticides, Extraction, Wastewater, COSMO-RS, Thermophysical charactaristics

## Abstract

Insecticides pose hazardous environmental effects and can enter the food chain and contaminate water resources. Ionic liquids (ILs) have recently drawn much interest as environmentally friendly solvents and have been an efficient choice for extracting pesticides because of their outstanding thermophysical characteristics and tunable nature. In this study, ILs were screened using COSMO-RS (Conductor-like Screening Model for Real Solvents) to extract organochlorine insecticides from water at 289 K. A total of 165 ILs, a combination of 33 cations with five anions, were screened by COSMO-RS to predict the selectivity and capacity of the organochlorine insecticides at infinite dilution. The Organochlorine insecticide compounds, such as benzene hexachloride (BHC), Heptachlor, Aldrin, Gamma-Chlordane (γ-Chlordane), Endrin, and Methoxychlor are selected for this study. Charge density profiles show that Endrin and Methoxychlor compounds are strong H-bond acceptors and weak H-bond donors, while the rest of the compounds are H-bond donors with no H-bond acceptor potential. Moreover, it has been shown that ILs composed of halides and heteroatomic anions in conjunction with cations have enhanced selectivity and capacity for insecticides. Moreover, the hydrophobic phosphonium-based ILs have enhanced selectivity and capacity for insecticides. In BHC extraction, the selectivity of 1,3-dimethyl-imidazolium chloride was found to be the highest at 1074.06, whereas 2-hydroxyethyl trimethyl ammonium chloride exhibited the highest capacity being 84.0.1,3-dimethyl-imidazolium chloride exhibits the highest performance index, which is 57064.77. In addition, the ILs that have been chosen are well-recognized as environmentally friendly and very effective solvents to extract insecticides from water. As a result, this study evaluated that ILs could be promising solvents that may be further developed for the extraction of insecticides from contaminated water.

## 1 Introduction

The current use of pesticides in agriculture is a significant factor in the growth rates of agricultural production. The Environmental Protection Agency (EPA) defines pesticides as a class of chemical compounds used to manage and deter the population of pests; any plants, animals, or microorganisms that affect food, health, or comfort are considered pests. Pesticides are substances that control or eradicate noxious pests such as creatures, organisms that cause plant diseases, and weeds [1]. The term “pesticide” refers to a broad category of insecticides, fungicides, herbicides, garden chemicals, household disinfectants, and rodenticides used to kill and protect against pests [2]. Pesticides in groundwater resources harm humans and ecosystems; since they contain elements that can cause DNA deviations, those materials are likely mutagens [3]. The World Health Organization (WHO) estimates that acute poisoning from contact with pesticides affects about 100,000 people. The annual death rate ranges from 0.4 to 1.9% [2].

Because of their high resistance to environmental degradation, pesticides are likely to bioaccumulate to hazardous levels and cause health risks. These pesticides are part of the class of organochlorine pesticides (OCPs), which are the most widely used in agriculture due to their outstanding ability to protect crops [4]. Organochlorines have the ability to penetrate an organism's body through various ways, including the skin, and lungs, and are absorbed through the gut wall. Being exposed to these contaminants can lead to a variety of symptoms including headaches, nausea, dizziness, vomiting, tremors, impaired coordination, and mental confusion [5]. Pesticides can be divided into two main categories: synthetic and natural. The synthetic ones are categorized into inorganic and “organic-or chemical”, while the chemical ones are grouped into four types based on their sources:Carbamate, organophosphate, organochlorine, and pyrethroid [2].

Pesticides are found in the soil, surface water, and atmosphere. Contaminated soil and water allow these pesticides to enter the food chain. Besides their solubility in water, pesticides have detrimental effects on delicate non-target organisms like humans and animals. Therefore, it has become a significant environmental issue, and it is necessary to clean up pesticides. One type of pollution brought on by the incorrect application of pesticides is water contamination rendering it toxic and unfit for human consumption. This can happen when pesticidess are applied to agricultural land and carried into nearby water bodies by the wind and rain. Generally, pesticide levels in surface waterways are substantially greater than in groundwater, due to farm runoff and spray drift contamination. However, pesticides can seep into the ground from polluted surface water, be improperly disposed of, or leak or spill accidently [6]. Insecticides are considered the most toxic pesticides of all types [6]; since these chemicals are chlorinated hydrocarbons and have poor solubility in water, high persistence, high solubility in lipids, and low polarity [7]. Besides, this substance concentrates and builds up in the food chain. The structure of insecticides, as chlorinated hydrocarbons, has allowed them to linger in the environment and contaminate all life forms [8].

To overcome this issue, several techniques were applied for insecticides removal, such as adsorption [9], activated carbon [10], enzymatic treatment [11], membrane filtration [12], liquid-liquid extraction (LLE) [13], and Dispersive liquid-liquid microextraction (DLLME) [1]. LLE is an effective option for removing pesticides from water solutions. Recently, techniques utilizing aqueous biphasic systems (ABS) have gained attention because of their ability to exploit competition for water molecules and unique interactions between polymers and salts, making them an attractive option for several reasons. They exhibit low cost and energy demands, as well as quick phase separation. However, polymer phases are viscous, making ABSs challenging to operate during extraction. Furthermore, ABSs composed of two polymers or one polymer and an inorganic salt had limited phase polarities, limiting their application [14]. Besides, DLLME offers a rapid, straightforward, and highly efficient approach for various extraction studies, specifically for the extraction of pesticides like pyrethroids [15].

Several extractants are commonly used, such as chlorobenzene [16], dichloromethane [17], and dibromoethane [18]. These organic solvents are toxic, hazardous, flammable, and damaging to the environment [19]. Given the potential harm to the aquatic environment and the challenges associated with separating solvents in liquid-liquid extraction (LLE), it is important to address the issue of organic solvent contamination [20].

The term ionic liquid (IL), also referred to as a room temperature ionic liquid (RTIL), has emerged as a viable alternative for organic solvents in LLE [21]. Ionic liquids (ILs) are a new generation of organic IL salts recently introduced as a class of new, potentially green solvents. ILs are low-melting salts that combine to form liquids composed of ions [22]. Moreover, ILs are known as “designer” solvents because their physical characteristics, such as density, viscosity, and surface tension, may be altered to suit the needs of specific applications by the suitable combinations of cation and anion [23].

The effectiveness of using ILs and identifying viable synthetic targets depends on the structure, chemical, thermophysical, and physicochemical properties of ILs. Important properties for extraction processes include selectivity and capacity, which can be estimated directly for various separation issues from the activity coefficients at infinite dilution [24]. The choice of extractants heavily influences the activity coefficients at the solute's infinite dilution (AC^id^) in ILs. The computational techniques enable the easy handling of enormous amounts of data without losing critical information or wasting valuable time. Furthermore, it is considered advantageous and essential to comprehend the specifications and the interactions of molecules and different solvents and to reduce the cost and number of trials in experimental testing.

The Conductor-like Screening Model for Real Solvents (COSMO-RS) simulation tool estimates the thermodynamic properties of compounds and mixtures by combining quantum chemistry and statistical thermodynamics. In particular, COSMO-RS enables the estimation of the infinite dilution activity coefficient (AC^id^) for solutes, which provides the selectivity and solvent capacity of significant ILs with non-available experimental data for a specific extraction application [25]. Therefore, it is proved to be valuable and successful in designing solvents for desulfurization [26], denitrification, alkane/aromatic and aromatic/aromatic separation, and solubility studies of drugs in aqueous solution [27,28]. As a result, a COSMO-RS software package is an efficient tool for this study to choose a potential ionic liquid to extract Organochlorine insecticides from water.

For the first time, COSMO-RS is used to study the chemical nature of insecticides compounds and assess the selectivity and capacity of ILs to extract organochlorine insecticides from water. Benzene hexachloride (BHC), heptachlor, aldrin, gamma-chlordane (γ-Chlordane), endrin, and methoxychlor were chosen for this study. Table 1 provides the insecticides' classification and category [29]. However, 33 cations were used to study the impact of cation hetero-aromaticity, aromaticity, alkyl chain length, and anion nature, along with five anions. A number of these cations were aromatic-based, including 1-butyl-3-methyl imidazolium [C_4_mim]^+^, 1-ethyl-4-methyl-pyridinium [E_MPY_]^+^, and non-aromatic-based cation 1-octyl-1-methyl-pyrrolidinium [C_8_M_PYR_]^+^, methyl-tributyl-ammonium [N_1_,_4_,_4_,_4_]^+^, propyl-cholinium [N_1_,_1_,_3_,_2_-OH]^+^, and tetra-N-butyl-phosphonium [P_4_,_4_,_4_,_4_]^+^, were chosen to study the removal of insecticides from water.

**Table 1:** The category and the classification of insecticides compounds.

Compound name	Category	Class	References
• Benzene hexachloride (BHC)	Insecticides	Organochlorines (used to control disease vectors in an urban environment)	[30]
• Heptachlor			
• Aldrin			
• Gamma-Chlordane (γ-Chlordane)			
• Endrin			
• Methoxychlor			

The aim of this study is to develop ILs for extracting various insecticides from surface water using an efficient treatment method, allowing for the recycling and reuse of large amounts of wastewater. In addition, the study deliverables are anticipated to assist in applied research and contribute to a long-term research vision. Furthermore, it is important to focus on advancing scientific knowledge in synthesizing complex solvents and their practical use in water treatment. Also, it discusses the correlation between ILs and the organic components in the aqueous phase. This study will greatly enhance the researcher's understanding of research techniques in advanced materials and wastewater treatment processes.

## 2 Materials

The category and classification of insecticides used in this study are shown in Table 1, while the cations and anions used in this study are given in Table 2, Table 3, respectively.

**Table 2:** List of cations studied.

Sr no.	Cations	Abbreviations	Structure
1	1,2-dimethyl-3-ethyl-imidazolium	[C_2.1_mim]^+^	
2	1,3-dimethyl-imidazolium	[C_1_mim]^+^	
3	1-butyl-3-methyl-imidazolium	[C_4_mim]^+^	
4	1-butyronitrile-2,3-dimethyl-imidazolium	[C_p_Mmim]^+^	
5	1-butyronitrile-3-methyl-imidazolium	[C_p_mim]^+^	
6	1-ethyl-3-methyl-imidazolium	[C_2_mim]^+^	
7	1-methyl-3-propyl-imidazolium	[C_3_mim]^+^	
8	1-methyloxymethyl-3-methyl-imidazolium	[C_2_Omim]^+^	
9	2,3-dimethyl-1-hexyl-imidazolium	[HMmim]^+^	
10	2-hydroxyethyl-trimethyl-ammonium	[Ch]^+^	
11	Ethyl-dimethyl-propyl-ammonium	[N_2,1,1,3_]^+^	
12	Methyl-tributyl-ammonium	[N_1,4,4,4_]^+^	
13	Methyl-tri octyl-ammonium	[N_1,8,8,8_]^+^	
14	tetra-N-butyl-ammonium	[N_4,4,4,4_]^+^	
15	Trimethyl-butyl-ammonium	[N_4,1,1,1_]^+^	
16	1-benzyl-3-methyl-pyridinium	[_1,3_Bzmpy]^+^	
17	1-butyl-2-methyl-pyridinium	[_1,2_Bmpy]^+^	
18	1-butyl-3-methyl-pyridinium	[_1,3_Bmpy]^+^	
19	1-butyl-4-methyl-pyridinium	[Bmpy]^+^	
20	1-ethyl-4-methyl-pyridinium	[Empy]^+^	
21	1-propyl-4-methyl-pyridinium	[C_3_mpy]^+^	
22	Ethyl-pyridinium	[Epy]^+^	
23	1-benzyl-1-methyl-pyrrolidinium	[BzMpyr]^+^	
24	1-butyl-1-methyl-pyrrolidinium	[C_4_Mpyr]^+^	
25	1-octyl-1-methyl-pyrrolidinium	[C_8_Mpyr]^+^	
26	1-pentyl-1-methyl-pyrrolidinium	[C_5_Mpyr]^+^	
27	1-propyl-1-methyl-pyrrolidinium	[C_3_Mpyr]^+^	
28	tetra-N-butyl-phosphonium	[P_4,4,4,4_]^+^	
29	tributyl-tetradecyl-phosphonium	[P_4,4,4,14_]^+^	
30	Tri hexyl-tetradecyl-phosphonium	[P_6,6,6,14_]^+^	
31	Tri isobutyl-methyl-phosphonium	[P_1,4,4,4_]^+^	
32	methyl-tri octyl-phosphonium	[P_1,8,8,8_]^+^	
33	Propyl-cholinium	[N_1,1,3,2_-OH]^+^

**Table 3:** List of anions studied.

Sr.no.	Anions	Abbreviation	Structure
1	Chloride	[Cl]^-^	
2	Bromide	[Br]^-^	
3	Iodide	[I]^-^	
4	tris-trifluoro-methyl-sulfonyl-methanide	mFAP	
5	bis-pentafluoro-ethyl-sulfonyl-imide	BEI	

## 3 Methodology

### 3.1 Computational details

#### 3.1.1 Molecular electrostatic potential

Understanding the electrophilic and nucleophilic reaction regions is crucial in examining biological coupling analysis and hydrogen bonding interactions. MEP was computed to identify the reactive regions (nucleophilic and electrophilic) in the examined ILs. This information enables us to identify the relative polarity for the optimized geometry. In addition, COSMO-RS was utilized to determine the classification of the molecules as either hydrogen bond donors (HBD) or hydrogen bond acceptors (HBA).

The first step in using COSMO-RS to predict thermodynamic properties is to generate optimized chemical structures of interacting species. ChemSketch and GaussView 6.0 were initially used to draw the chemical species' structures. The Gaussian-09 software package was used to perform the quantum calculations for optimization and energy calculation, and the electrostatic potential map (ESP) was obtained to study the charge distribution. DFT-level quantum calculations were done; similar calculations were performed elsewhere on various compounds [[31], [32], [33], [34]]. For optimization and energy calculations, B3LYP- 6–31 + g(d,p), 6–311++g(d,p), 6–31g(d,p), 6–311 + g(d,p), 6–31g(d), and 6–311g(d,p) basis sets were employed to optimize the molecules of BHC, heptachlor, aldrin, gamma-chlordane, endrin, and Methoxychlor, respectively. The estimated sigma charge density, known as the sigma profile, and the sigma potential were obtained using COSMO-RS.

#### 3.1.2 Activity coefficients, selectivity, and capacity

The activity coefficients of insecticides and water in ILs at infinite dilution were predicted using COSMO-RS to calculate the selectivity and capacity of ILs at infinite dilution. Solvent extraction was considered the economical method for extracting insecticides from water. The selectivity and the capacity at infinite dilution are the two critical parameters to evaluate the extractive strength of the solvent. Selectivity is the degree to which a solvent prefers a specific solute, represented by the ratio of the compositions of solutes in ILs, as shown in equation (1):

(1) $$S_{12}^{\infty }={\left(\frac{\gamma_{2}^{\infty }}{\gamma_{1}^{\infty }}\right)}^{Ionic\;liquid\;phase}$$

where $\gamma_{2}^{\infty }$ is the activity coefficient of water at infinite dilution, while $\gamma_{1}^{\infty }$ is the activity coefficient of insecticides at infinite dilution. Capacity at infinite dilution represents the maximum quantity of the solute species that might be dissolved by the solvent, which is defined using equation (2):

(2) $$C^{\infty }={\left(\frac{1}{\gamma_{1}^{\infty }}\right)}^{Ionic\;liquid\;phase}$$

The effectiveness of the solvent that is related to the extraction of insecticides can be determined by calculating the performance index using equation (3) as given below:

(3) $$P.I={\left(\frac{\gamma_{2}^{\infty }}{{{(\gamma }_{1}^{\infty })}^{2}}\right)}^{Ionic\;liquid\;phase}$$

**Fig. 1:**
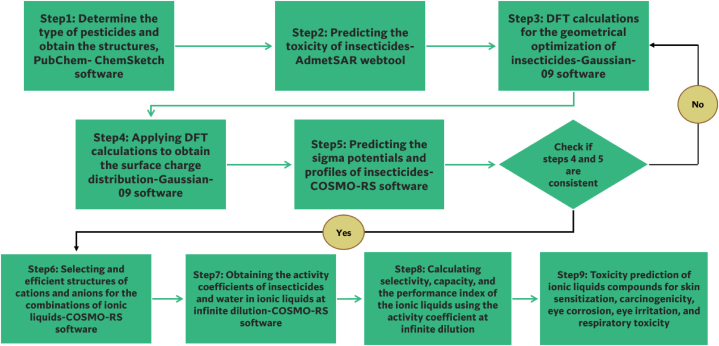
The protocol for screening ionic liquids to extract insecticides.

The protocol for screening ionic liquids to extract insecticides is depicted in Fig. 1.

### 3.2 Eco-toxicity assessment

The ADMET properties of environmental chemicals are crucial factors in drug discovery and environmental hazard assessment and are especially useful when conducting human hazard assessment. These properties include absorption, distribution, metabolism, excretion, and toxicity. The toxicity of ILs was evaluated using the AdmetSAR prediction tool, which utilized canonical smiles from PubChem and the Chemskitch software smiles generator tool for compound identification. It is essential to assess the potential toxicity of the ILs being studied in order to understand their effects on human health and ensure safe handling of these chemicals. The predicted impacts of ILs include skin sensitization, carcinogenicity, eye corrosion, eye irritation, and respiratory toxicity. The admetSAR server, also known as the ADMET structure-activity relationship server, is a valuable resource that provides extensive information and tools for predicting the ADMET properties of drug candidates and environmental chemicals [35,36]. The server contains over 200,000 ADMET annotated data points, manually curated from a wide range of large literature sources. These data points cover approximately 96 thousand unique compounds. The admetSAR server offers a user-friendly interface that allows users to conveniently search for chemical profiles using CASRN, common name, and similarity search.

## 4 Results and discussions

### 4.1 Validation of COSMO-RS predictions on commercial compound

**Fig. 2:**
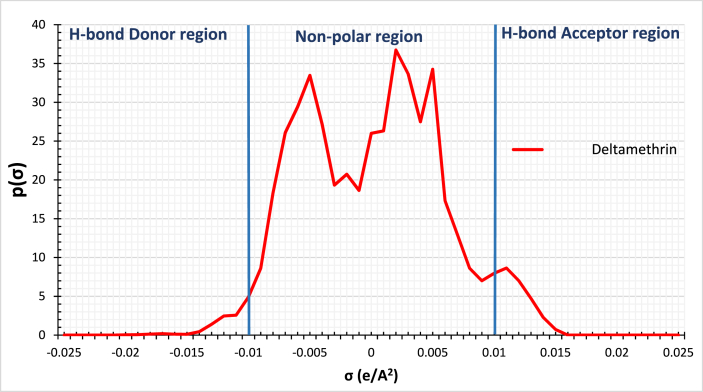
Sigma profile of Deltamethrin using COSMO-RS.

**Fig. 3:**
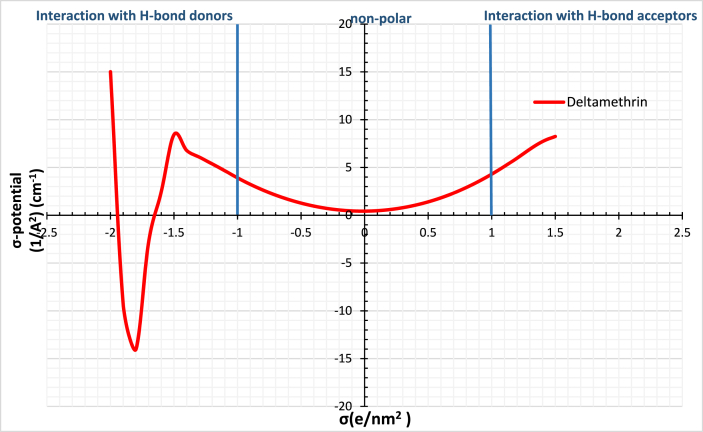
Sigma potential of Deltamethrin using COSMO-RS.

The prediction of the activity coefficient at infinite dilution of ILs is applied to commercial insecticides. Deltamethrin is one type of insecticide; it is a representative chemical of pyrethroids. The structure of Deltamethrin is presented in Table 4; pyrethroids are considered one of the most extensively used insecticides as agricultural insecticides for cotton, vegetables, and rice [37,38]. Similar calculations using COSMO-RS are performed to extract Deltamethrin. Deltamethrin possesses two peaks on the Sigma profile, within the negative and the positive regions at −0.012 e/A^2^ and 0.011 e/A^2^, respectively, represented in Fig. 2. Also, Deltamethrin shows an increasing trend toward the interaction with the hydrogen bond donors, while it shows a decreasing trend towards the interaction with hydrogen bond acceptors. This observation indicates that Deltamethrin acts as a strong H-bond acceptor, as shown in Fig. 3.

**Table 4:** The chemical configuration and the surface charge distribution of Deltamethrin. Nitrogen is indicated by blue, while Oxygen and Bromine are indicated by red and Dark red, respectively.

Compound name	Chemical configuration	Surface charge distribution
Deltamethrin

**Fig. 4:**
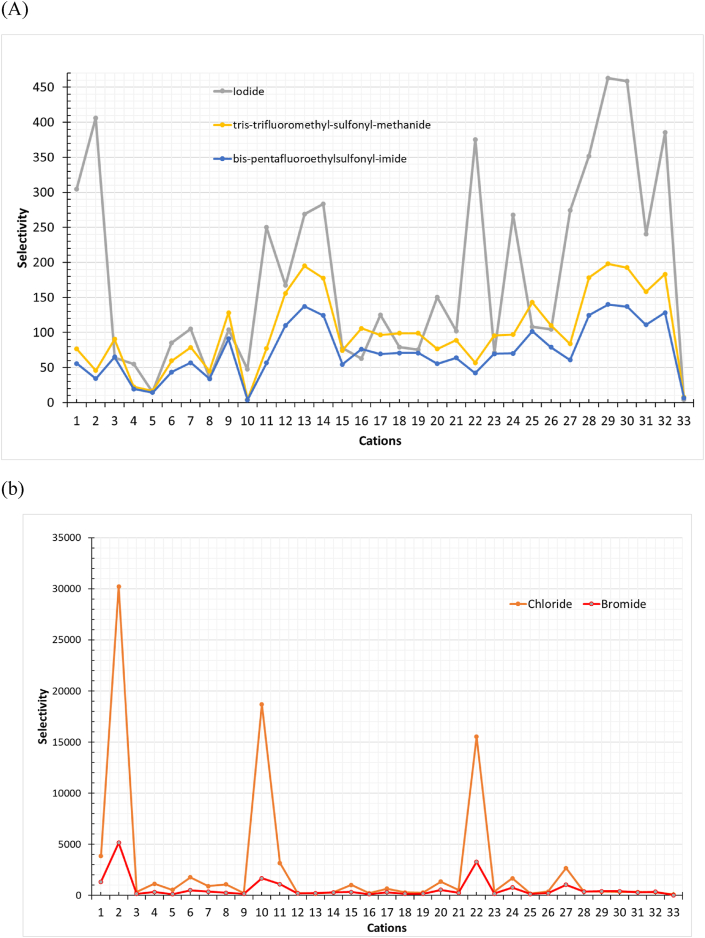
Selectivity of (a) ILs consists of Iodide, tris-trifluoro-methyl-sulfonyl-methanide, and bis-pentafluoro-ethyl-sulfonyl-imide and (b)ILs consists of chloride and bromide towards Deltamethrin. (Cation number refers to the identification key shown in Table 2).

**Fig. 5:**
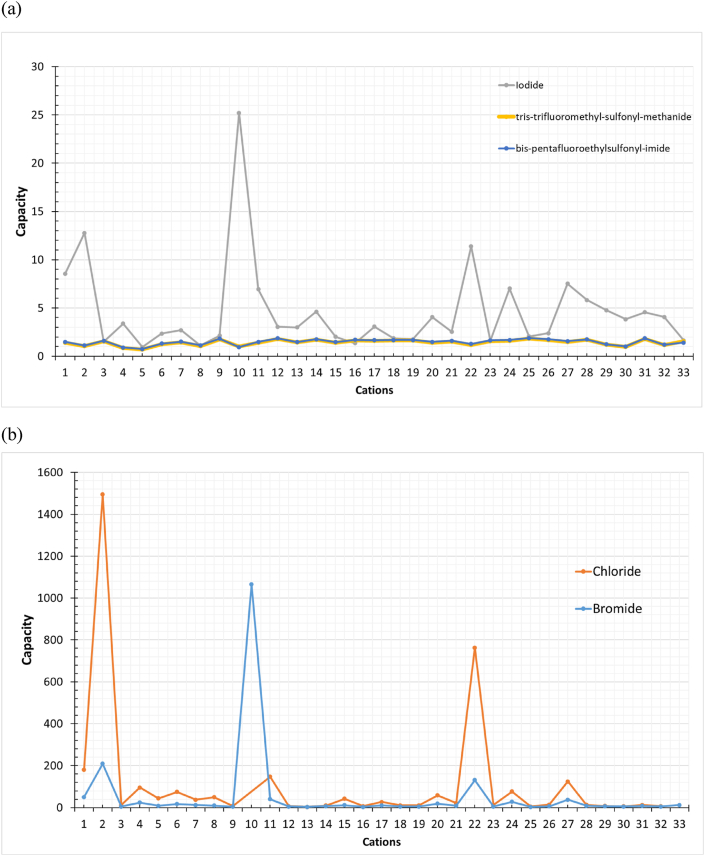
Capacity of (a) ILs consists of Iodide, tris-trifluoro-methyl-sulfonyl-methanide, and bis-pentafluoro-ethyl-sulfonyl-imide and (b)ILs consists of chloride and bromide towards Deltamethrin. (Cation number refers to the identification key shown in Table 2).

**Fig. 6:**
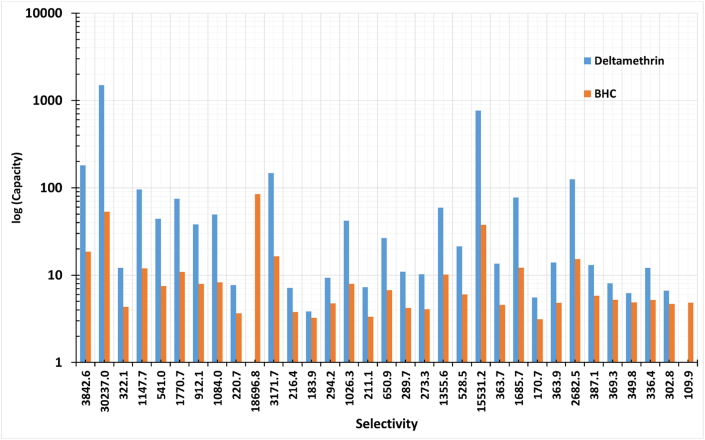
Log(capacity) against the selectivity of ILs consisting of chloride for Deltamethrin, indicated Blue, compared to BHC, indicated Orange. (For interpretation of the references to color in this figure legend, the reader is referred to the Web version of this article.)

Moreover, the anions were screened over the selected cations to extract Deltamethrin. ILs that consist of chloride and bromide show high selectivity and capacity, as shown in Fig. 4 (a,b) and Fig. 5 (a,b), respectively. Besides, Fig. 6 shows higher capacity values related to selectivity values represented by the ILs for Deltamethrin than those for BHC; since deltamethrin is a strong H-bond acceptor; due to the presence of oxygen and heteroatoms in its structure. Consequently, the interaction with the ILs increases as Deltamethrin provides an electron pair closer to the behavior of Methoxychlor and Endrin. Deltamethrin shows its chemical nature by being a strong H-bond acceptor. On the other hand, Methoxychlor and Endrin possess an affinity to be weak H-bond donors and H-bond acceptors. In addition, these results reflect the proper selection of the ILs combinations in which the selection is compatible with insecticides with types other than organochlorine.

### 4.2 Sigma profiles and sigma potentials of insecticides

**Fig. 7:**
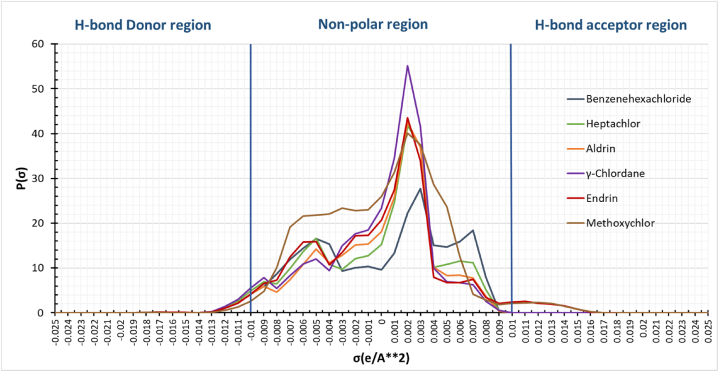
Sigma profiles of the organochlorine insecticides.

**Fig. 8:**
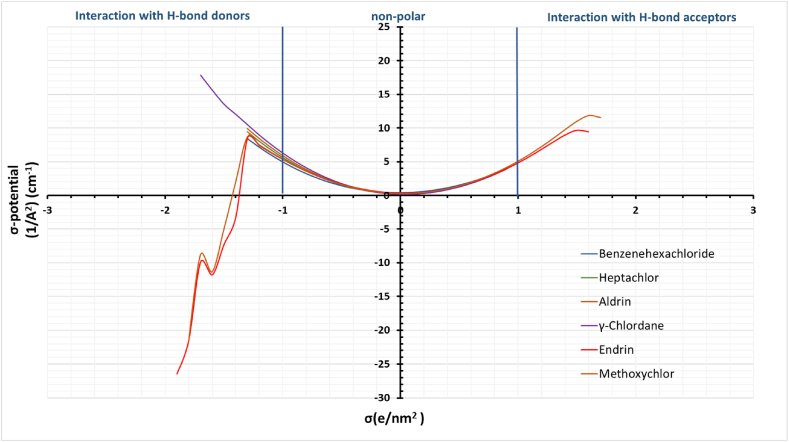
Sigma potentials of the organochlorine insecticides.

The σ-profile of model compounds is displayed in Fig. 7. The x-axis represents the screening charge density (σ) of the organochlorine insecticides surface segment, whereas the y-axis represents p(σ)A, where p(σ) is the probability of a surface segment having a screening charge density σ [39]. The sigma potentials of organochlorine insecticides reveal their response to a molecular surface of a given polarity. The sigma potentials of the organochlorine at 298.15 K are displayed in Fig. 8. The figure shows two significant peaks in the positive polar regions for Endrin, and Methoxychlor indicated Red, and Brown, respectively. The peaks at the positive side of the profile represent the polarized charge at 0.011 e/A^2^, which depicts the Oxygen and Chlorine atoms portion in both Endrin and Methoxychlor, indicating its ability to act as a hydrogen bond acceptor. As a result, the model compounds are attracted toward the hydrogen bond donor group. Therefore, they have an increasing trend in their interaction with hydrogen bond donor molecules, as shown in the σ-potential histogram in Fig. 8.

Moreover, Hydrogen atoms in the insecticides show peaks within the negative region at (−0.011, −0.012) e/A^2^, depicting their ability to act as hydrogen bond donors, as in Fig. 7. Hence, it has a decreasing trend towards the hydrogen bond acceptor species, as evident from the σ-potential histogram in Fig. 8. As a result, Endrin and Methoxychlor compounds reflect their chemical properties, becoming strong H-bond acceptors and weak H-bond donors. In contrast, the other compounds show their ability to act as hydrogen bond donors with no possibility of being an H-bond acceptor.

The distribution of charges on a surface in Table 5 is represented by color, with red representing the negative (-ve) charge, while blue and green represent the positive (+ve) and neutral (0) charge, respectively. This information is crucial in determining the potential of IL as an insecticide removal solvent.

**Table 5:** The surface charge distribution of the organochlorine insecticides.

Compound name	Chemical configuration	Surface charge distribution
Benzene hexachloride		
Heptachlor		
Aldrin		
Gamma-Chlordane		
Endrin		
Methoxychlor		

### 4.3 Selectivity of ionic liquids

**Fig. 9:**
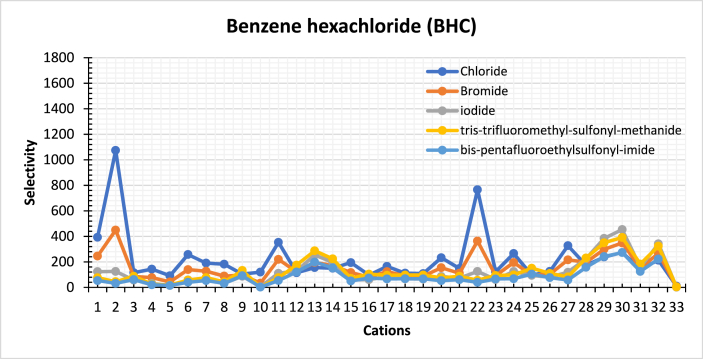
Calculated selectivity of various ILs towards BHC at 298 K in water (Cation number refers to the identification key shown in Table 2).

The selectivity towards insecticide compounds with 33 cations, including imidazolium, pyridinium, pyrrolidinium, phosphonium, ammonium, and choline-based cations in combination with five different anions, was calculated using COSMO-RS. BHC is chosen to represent the findings since the chemical nature of the compounds investigated is similar. Fig. 9 shows the selectivity of these various ILs toward BHC. The selectivity of the other compounds is shown in Fig. S-1. The numbers of cations and anions are identified earlier in Table 2, Table 3, respectively.

Khan et al. investigated several combinations of ILs and observed that some combinations had an activity coefficient of water greater than one, indicating a positive deviation to the ideality. This finding suggests that water exhibits weak interactions with ILs [40]. Nevertheless, our analysis yielded comparable findings for the activity coefficient of water at infinite dilution, indicating activity coefficients above unity. Consequently, this will result in weak interaction between water and ILs. At the same time, insecticides deliver strong interaction with ILs, as evidenced by the results in Fig. 9.

Furthermore, the presence of a ring structure in ILs, particularly those containing cations such as imidazolium and pyridinium, leads to a reduction in selectivity. The reason for this decrease is the delocalized electrons, which hinder the π -π interactions. Moreover, as the aromatic rings of the cation increase, the aromaticity decreases; due to the additional rings that will hinder the π-π interaction and will not engage in the intramolecular interaction. Furthermore, it is observed that the selectivity increases with the increase of the heteroatoms in the aromatic rings present in the aromatic cations and anions, enhancing the interaction ability of the cation. On the other hand, ammonium and phosphonium-based cations show higher selectivity [41].

In addition, the interaction between the ILs and insecticides is governed by several factors that affect the selectivity at infinite dilution to the heteroatoms present in the molecules; these factors are.

a) Hydrogen bonding between cation and heteroatoms of the insecticides
b) Hydrogen bonding between anions, halides (Cl^−^, Br-and I^−^), and the hydrogen attached to the insecticides
c) Alkyl chain length of the cation
d) The dipole moment of the ILs [42,43].

#### 4.3.1 Effect of anions

**Fig. 10:**
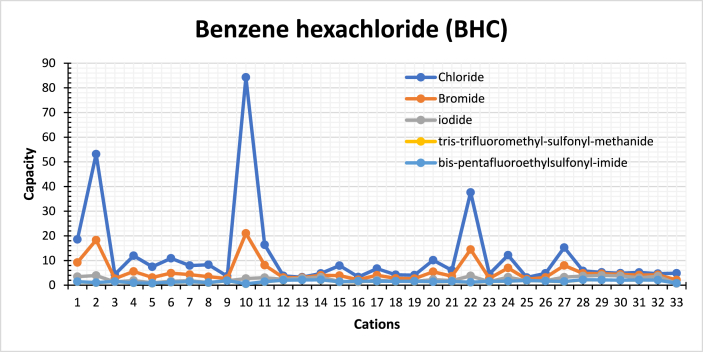
Calculated capacity of various ILs towards BHC compounds at 298 K in water (Cation number refers to the identification key shown in Table 2).

Initially, an estimation of 41 anions combined with 1,2-dimethyl-3-ethyl-imidazolium for the extraction of insecticides using COSMO-RS is implemented to evaluate the effectiveness of different anions, see Tables S–1. However, after investigating vast combinations of ILs, the anions were refined to five compounds with the highest selectivity and capacity values, as presented in Fig. 9, Fig. 10, respectively.

Several anions, such as dicyanamide, docusate, dodecyl benzene sulfonate, formate, and glycinate, were screened over 1,2-dimethyl-3-ethyl-imidazolium, see Tables S–1. The combinations of ILs based on the mentioned anions deliver weak selectivity and capacity values toward insecticides. On the other hand, halides Cl^−^, Br^−^, and I^−^, provide the best selectivity values in the order Cl^−^ > Br^−^ > I^−^; this is due to the high electronegativity related to these anions. Besides, the electron affinity also affects the order in that Cl^−^ has less electron affinity than Br^−^, and Br^−^ is also less than I^−^, which makes Cl^−^ show the best selectivity.

Furthermore, tris-trifluoro-methyl-sulfonyl-methanide and bis-pentafluoro-ethyl-sulfonyl-imide show high selectivity values due to the number of heteroatoms available in these compounds. Also, the presence of Fluorine will improve the interaction of insecticides by enhancing hydrogen bonding. As a result, the solubility of insecticides will be improved. Moreover, the fluorination of the anion reduces the strength of the interaction between water and the ionic liquid. This is evident from the high values of the activity coefficient of water when the anion is fluorinated [40].

In addition, the ILs consisting of Cl-, Br-, I-, tris-trifluoro-methyl-sulfonyl-methanide, and bis-pentafluoro-ethyl-sulfonyl-imide show the best selectivity and capacity among the other combinations. The ILs result in an activity coefficient of water higher than unity, indicating a weak interaction between water and ILs; these combinations of ILs will deliver high affinity to insecticides. As a result, there is substantial interaction between insecticides and ILs, as evidenced by insecticide activity coefficients that are less than unity, see Tables S–2.

#### 4.3.2 Effect of cations

This study included 33 cations-based imidazolium, pyridinium, pyrrolidinium, ammonium, phosphonium, and choline. Cations were screened over the selected anions to extract insecticides from water using COSMO-RS. The Selectivity of ILs towards the insecticides is observed to follow a certain sequence taking BHC as an example as follow:

BHC: Phosphonium > Ammonium > Pyrrolidinium > Imidazolium > Pyridinium > Cholinium.

The cations that do not contain aromatic rings in their structure are clear of π-electron cloud. The absence of the delocalized electrons will reflect positively on the interaction of the ILs with the insecticides and provide more tendency to provide electrons and enhance the π- π interactions. Thus, the selectivity will rise towards insecticides, resulting in selectivity values higher than imidazolium and pyridinium. This will result in a better interaction with the compounds.

The increase of heteroatoms will increase the selectivity, increasing the ability of anion to interact by increasing the hydrogen bonding, giving better solubility properties for insecticides. Also, this refers to the steric hindrance of the Imidazolium and pyridinium to the compounds, which will drop the selectivity and reduce the hydrogen bonding strength between the cation and the insecticides. Furthermore, increasing the heteroatoms in the ring for cations-based will raise the selectivity and capacity and will decrease the hindrance of the cations containing aromatic rings.

### 4.4 Capacity of ionic liquids

The capacity of ILs towards the insecticides is observed to follow a certain sequence taking BHC as an example as follow: BHC: Imidazolium > Pyridinium > Pyrrolidinium > Ammonium > Phosphonium > Cholinium. Cations-based Imidazolium and cation-based phosphonium have higher capacity; since the capacity increases as the π electron cloud of cations decreases. Besides, the interaction of the cation takes place through the hydrogen bonding between the cation and the heteroatoms of the insecticides. The capacity of different combinations of ILs towards BHC is presented in Fig. 10. The capacity values related the rest of the compounds is shown in Fig. S-2 shows.

Furthermore, increasing the heteroatoms in the molecule enhances the charge sharing of cations; hence, the cation interaction ability increases with the insecticides. Also, the number of heteroatoms in the cation ring enhances ILs' capacity. The capacity of the imidazolium cation containing two nitrogen atoms is greater than the pyrrolidinium cation. This may refer to the inductive effect of [B_mim_]^+^, higher than the other cations, shifting the electron cloud towards the more electronegative atoms, the two nitrogen atoms. In addition, it is observed that solvents with high selectivity have a low capacity for the solute, so generally, a high selectivity corresponds to low capacity values.

### 4.5 The effect of alkyl chain length on selectivity and capacity

**Fig. 11:**
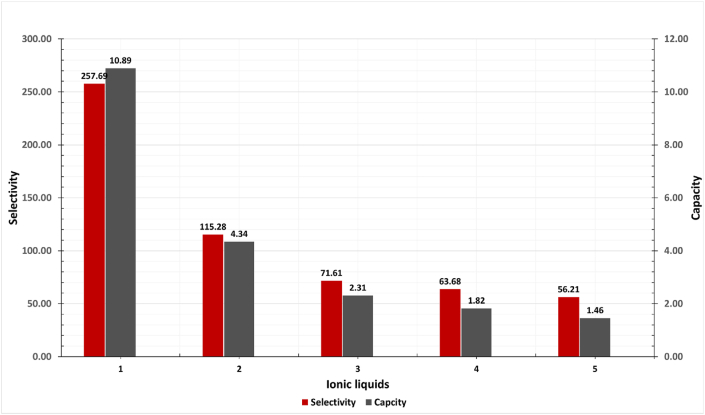
The effect of imidazolium cation alkyl chain length on the selectivity of ILs for insecticides compounds at 298 K. (The number of ILs refers to the identification key shown in Table 7).

The effect of the alkyl chain length of the imidazolium-based cations was studied by varying the length from ethyl to nonyl. The selectivity is depicted in Fig. 11 and detailed in Table 6.

**Table 6:** The effect of imidazolium cation alkyl chain length on the selectivity and capacity of ILs for insecticides compounds at 298 K.

Sr no.	IL based imidazolium	Selectivity	Capacity
**1**	1-ethyl-3-methyl-imidazolium chloride	257.69	10.89
**2**	1-butyl-3-methyl-imidazolium chloride	115.28	4.34
**3**	1-hexyl-3-methyl-imidazolium chloride	71.61	2.31
**4**	1-octyl-3-methyl-imidazolium chloride	63.68	1.82
**5**	1-nonyl-3-methyl-imidazolium chloride	56.20	1.46

The infinite dilution selectivity changed significantly, especially from 1-ethyl-3-methyl-imidazolium chloride to 1-hexyl-3-methyl-imidazolium chloride. The increase in alkyl chain length decreases the selectivity and the capacity of ILs due to the decrease in the charge density of the cations. The growth of the cation alkyl chain length will increase the molar volume and reduce the charge density, and affect the movement of the ILs, owing to increased twisting and tangling of alkyl chains, making it difficult for molecules to interact. As the alkyl chain lengthens, the size of the cation increases, increasing the volume of the IL and limiting its ability to move freely due to its bulky structure [44]..

**Table 7:** Toxicity prediction of ILs compounds for skin sensitization, carcinogenicity, eye corrosion, eye irritation, and respiratory toxicity.

Property	Skin sensitization	Carcinogenicity	Eye corrosion	Eye irritation	Respiratory toxicity
IL	1,2-dimethyl-3-ethyl-imidazolium Chloride				
Probability	0.85 (nontoxic)	0.84 (nontoxic)	0.94 (nontoxic)	0.62 (toxic)	0.56 (toxic)
IL	1,3-dimethyl-imidazolium Chloride				
Probability	0.84 (nontoxic)	0.81 (nontoxic)	0.67 (toxic)	0.91 (toxic)	0.54 (nontoxic)
IL	2-hydroxyethyl-trimethyl-ammonium Chloride				
Probability	0.7 (nontoxic)	0.6 (nontoxic)	0.62 (toxic)	0.99 (toxic)	0.84 (toxic)
IL	Ethyl-pyridinium Chloride				
Probability	0.58 (nontoxic)	0.57 (nontoxic)	0.55 (toxic)	0.97 (toxic)	0.81 (nontoxic)

In addition, the capacity reduction refers to the increase of the compactness between the cation and the anion with the increase of the alkyl chain length, which will reduce the absorption area and weaken the electrostatic interaction between the anion or cation and insecticides [45].

#### 4.5.1 Performance index (P.I)

**Fig. 12:**
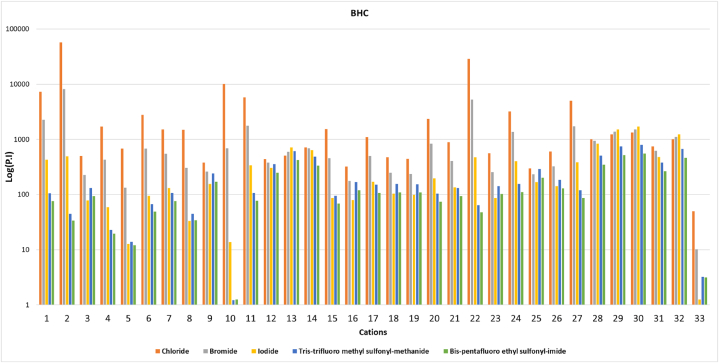
The performance index (P.I) of ILs for BHC. **(**Cation number refers to the identification key shown in Table 2).

A crucial point for selecting the solvent used for extraction is being economically feasible. The proper selection should interact well with consuming in appropriate amounts to satisfy the economic consideration. This refers to solvents that deliver distinguished properties in terms of providing high selectivity with high capacity values, and this results in an efficient solution for industrial applications. The Performance Index (P.I) value can be employed to express the efficacy of any solvent. It is observed that there are statistically significant differences in the selectivity and capacity values. Thus, the P.I plays a key role in selecting the optimal combination with higher selectivity and relevant capacity compared to other combinations. Fig. 12 represents the P.I values of the studied combinations of ILs for extracting BHC. Fig. S-3 represents the investigated P.I values for the other insecticides compounds.

The highest P.I was obtained for imidazolium, pyridinium, and pyrrolidinium-based ILs because of their high selectivity and capacity values. It is observed that 1,3-dimethyl-imidazolium with chloride gave the highest selectivity value, i.e., 1074.06 for BHC. Besides, 2-hydroxyethyl trimethyl ammonium with chloride possesses the highest capacity, i.e., 84.0 for BHC. In addition, the highest performance index was obtained by combining 1,3-dimethyl-imidazolium with chloride, i.e., 57064.77 for BHC; since the high selectivity corresponds to a relatively high capacity.

## 5 Human health effects of insecticides and ILs on living organisms

Insecticides compounds are biologically active by nature, and many may be harmful. Previous studies show insecticide metabolites are frequently detected in groundwater at higher amounts than parent substances from agricultural and amenity use. A broad scale of use is commonly associated with products grown in large areas, such as potatoes or maize, or with active compounds utilized for various applications. Another concern is the soil's susceptibility to pesticide leakage into groundwater [46]. According to an estimate from World Health Organization (WHO) and the United Nations Environmental Program (UNEP), around 200,000 people die, and three million are poisoned by pesticides each year, with the great majority (95%) of cases occurring in the developed countries [30].

To overcome this issue, ILs are considered a greener alternative to the commonly used extractants [47], including chlorobenzene [16], dichloromethane [17], and dibromoethane [18] that are employed to extract insecticides. The conventional organic solvents mentioned possess toxicity, hazardous properties, flammability, and have the potential to cause environmental pollution [19]. The probabilities of eco-toxicity evaluation parameters of the studied ILs were predicted using admetSAR. The toxicity assessment reveals that the eco-friendly parameters listed in Table 7 have different levels of safe consequences. For example, 1.2-dimethyl-3-ethyl-imidazolium Chloride is non-toxic for eye corrosion by 0.94 out of 1. Overall, ILs have been shown to be non-carcinogenic and do not have any harmful effects on the skin.

## 6 Conclusion

The main objective of this study is to develop ILs for the extraction of different insecticides from surface water using COSMO-RS. The COSMO-RS is used to estimate the selectivity and capacity of ILs towards insecticides at infinite dilution. In this study, 165 combinations that consist of 5 anions and 33 cations are investigated, and the following is concluded.

• The results show that Halides, Cl-, Br-, and I- possess the highest selectivity. This is due to the high electronegativity. However, less electron affinity of the halides shows higher selectivity for insecticides, following the trend: Insecticides: Cl^−^ > Br-> I^−^. However, the relation of ionic liquid selectivity is directly proportional to its capacity.
• The cations with no aromatic rings have higher selectivity. Moreover, the interaction ability of cations increases as the heteroatoms increase. Hence, this will enhance the charge sharing, improve the cation's interaction ability, increase the hydrogen bonding, and provide better insecticide solubility properties. Besides, increasing heteroatoms in cation will increase its selectivity towards the insecticides.
• Increasing the length of the alkyl chain increases its volume and affects the movement of the ILs due to the increased twisting and tangling of the alkyl chains, making it difficult for molecules to interact. Thus, this will decrease selectivity.
• Overall, ILs are considered green and efficient solvents for extracting insecticides due to their high chemical and thermal stability, low volatility, low melting point, and targeted rheological characteristics.
• It was found that COSMO-RS has limitations in terms of adding some anions structures to the software platform. However, no such issues were found for cations structures.
• Additional research can be conducted to explore the distribution coefficient and extraction efficiency of the ILs. Employing ILs in combination with other technologies, like membranes, can be studied to enhance the efficiency of the separation process.

## Data availability

No data availability. The data that has been used is confidential.

## CRediT authorship contribution statement

**Mohammad K. Al Hassan:** Writing – original draft, Validation, Software, Methodology, Investigation, Formal analysis. **Mustafa S. Nasser:** Writing – review & editing, Supervision, Project administration, Methodology, Funding acquisition. **Ibnelwaleed A. Hussein:** Writing – review & editing, Supervision, Conceptualization. **Muneer Ba-Abbad:** Writing – review & editing. **Imran Khan:** Writing – review & editing, Conceptualization.

## Declaration of competing interest

The authors declare that they have no known competing financial interests or personal relationships that could have appeared to influence the work reported in this paper.
