# Supplementary material for: Computational study on organochlorine insecticides extraction using ionic liquids
Source: Heliyon. 2024 Feb 12;10(4):e25931. doi: 10.1016/j.heliyon.2024.e25931 (PMC10884451; doi:10.1016/j.heliyon.2024.e25931)
Supplement: Multimedia component 1 [file mmc1.docx]

**Extraction of Organochlorine Insecticides from Contaminated Water by Using Ionic Liquid -A computational study (Supplementary Data)**

Mohammad K. Al Hassan^a^, Mustafa S. Nasser^a,b*^, Ibnelwaleed A. Hussein^a,b*^_,_

Muneer Ba-Abbad^a,b^, Imran Khan^c^

*^a^ Gas Processing Center, College of Engineering, P.O. Box 2713, Qatar University, Doha, Qatar*

*^b^ Chemical Engineering Department, College of Engineering, P.O. Box 2713, Qatar University, Doha, Qatar*

*^c^ Department of Chemistry, College of Science, Sultan Qaboos University, Muscat, Oman*

^*^Corresponding authors’ emails: [m.nasser@qu.edu.qa](mailto:m.nasser@qu.edu.qa) ; [ihussein@qu.edu.qa](mailto:ihussein@qu.edu.qa)

**Table S-1**: Sample of calculations of ILs consist of different anions combined to 1,2-dimethyl-3-ethyl-imidazolium to extract BHC from water at 298 K.

| IL | AC-Water | AC-BHC | Selectivity BHC | Capacity BHC |
| --- | --- | --- | --- | --- |
| 1,2-dimethyl-3-ethyl-imidazolium 2-2-methoxyethoxy-ethylsulfate | 0.391889 | 0.9030 | 0.4339495 | 1.107326 |
| 1,2-dimethyl-3-ethyl-imidazolium acesulfamate | 0.119698 | 1.4623 | 0.0818533 | 0.683829 |
| 1,2-dimethyl-3-ethyl-imidazolium acetate | 0.000087 | 0.3645 | 0.0002398 | 2.743261 |
| 1,2-dimethyl-3-ethyl-imidazolium alanate | 0.000234 | 0.6785 | 0.0003454 | 1.473639 |
| 1,2-dimethyl-3-ethyl-imidazolium bis-2,4,4-trimethyl-pentyl-phosphinate | 0.000219 | 0.6192 | 0.0003538 | 1.614760 |
| 1,2-dimethyl-3-ethyl-imidazolium bis-pentafluoro-ethyl-sulfonyl-imide | 37.58863 | 0.7011 | 53.613314 | 1.426316 |
| 1,2-dimethyl-3-ethyl-imidazolium bromide | 26.65042 | 0.1085 | 245.51682 | 9.212490 |
| 1,2-dimethyl-3-ethyl-imidazolium chloride | 21.19078 | 0.0539 | 393.02274 | 18.54687 |
| 1,2-dimethyl-3-ethyl-imidazolium dicyanamide | 0.130396 | 1.7883 | 0.0729137 | 0.559170 |
| 1,2-dimethyl-3-ethyl-imidazolium docusate | 0.093545 | 0.4964 | 0.1884098 | 2.014109 |
| 1,2-dimethyl-3-ethyl-imidazolium dodecyl-benzenesulfonate | 0.130502 | 0.5055 | 0.2581178 | 1.977882 |
| 1,2-dimethyl-3-ethyl-imidazolium formate | 0.000285 | 0.2192 | 0.0013002 | 4.560989 |
| 1,2-dimethyl-3-ethyl-imidazolium glycinate | 0.000276 | 0.4727 | 0.0005858 | 2.115493 |
| 1,2-dimethyl-3-ethyl-imidazolium heptafluoro-butyrate | 0.042874 | 1.2613 | 0.0339908 | 0.792801 |
| 1,2-dimethyl-3-ethyl-imidazolium hexadecanate | 0.000190 | 0.5293 | 0.0003600 | 1.889119 |
| 1,2-dimethyl-3-ethyl-imidazolium hexafluoro-antimonate | 68.09185 | 3.9749 | 17.130357 | 0.251577 |
| 1,2-dimethyl-3-ethyl-imidazolium hexafluoro-phosphate | 33.24987 | 4.5630 | 7.2867769 | 0.219152 |
| 1,2-dimethyl-3-ethyl-imidazolium imino acetic acid-acetate | 0.020604 | 1.0000 | 0.0206042 | 0.999969 |
| 1,2-dimethyl-3-ethyl-imidazolium iodide | 35.66667 | 0.2886 | 123.56855 | 3.464538 |
| 1,2-dimethyl-3-ethyl-imidazolium isobutyrate | 0.000096 | 0.6833 | 0.0001407 | 1.463456 |
| 1,2-dimethyl-3-ethyl-imidazolium isoleucinate | 0.000312 | 0.8213 | 0.0003799 | 1.217582 |
| 1,2-dimethyl-3-ethyl-imidazolium lactate | 0.002374 | 0.8500 | 0.0027929 | 1.176463 |
| 1,2-dimethyl-3-ethyl-imidazolium levulinate | 0.000451 | 0.5601 | 0.0008058 | 1.785258 |
| 1,2-dimethyl-3-ethyl-imidazolium lysinate | 0.000366 | 0.7880 | 0.0004646 | 1.268999 |
| 1,2-dimethyl-3-ethyl-imidazolium mesylate | 0.019984 | 0.5252 | 0.0380469 | 1.903800 |
| 1,2-dimethyl-3-ethyl-imidazolium nitrate | 0.013088 | 0.6149 | 0.0212828 | 1.626112 |
| 1,2-dimethyl-3-ethyl-imidazolium  p-toluenesulfonate | 0.067599 | 0.9082 | 0.0744269 | 1.100990 |
| 1,2-dimethyl-3-ethyl-imidazolium penta-decafluoro-actanoate | 0.071812 | 0.7204 | 0.0996792 | 1.388043 |
| 1,2-dimethyl-3-ethyl-imidazolium propionate | 0.000092 | 0.5565 | 0.0001655 | 1.796673 |
| 1,2-dimethyl-3-ethyl-imidazolium saccharinate | 0.085084 | 1.2748 | 0.0667382 | 0.784376 |
| 1,2-dimethyl-3-ethyl-imidazolium sarcosinate | 0.000116 | 0.5177 | 0.0002246 | 1.931395 |
| 1,2-dimethyl-3-ethyl-imidazolium succinamate | 0.002371 | 0.9700 | 0.0024446 | 1.030918 |
| 1,2-dimethyl-3-ethyl-imidazolium taurinate | 0.007015 | 0.7978 | 0.0087934 | 1.253341 |
| 1,2-dimethyl-3-ethyl-imidazolium tetracyano-borate | 7.589497 | 1.8446 | 4.1143174 | 0.542106 |
| 1,2-dimethyl-3-ethyl-imidazolium tetrafluoro-borate | 3.328749 | 2.0919 | 1.5912293 | 0.478026 |
| 1,2-dimethyl-3-ethyl-imidazolium thiocyanate | 0.129569 | 1.2416 | 0.1043535 | 0.805385 |
| 1,2-dimethyl-3-ethyl-imidazolium tricyano-methanide | 1.398981 | 2.2460 | 0.6228735 | 0.445233 |
| 1,2-dimethyl-3-ethyl-imidazolium  trifluoro-acetate | 0.026977 | 1.8208 | 0.0148161 | 0.549206 |
| 1,2-dimethyl-3-ethyl-imidazolium trimethyl-acetate | 0.00010671 | 0.8159 | 0.0001307 | 1.225586 |
| 1,2-dimethyl-3-ethyl-imidazoliumtris-trifluoromethyl-sulfonyl-methanide | 54.9550 | 0.72059 | 76.263006 | 1.387734 |

**Table S-2**: Sample of calculations of ILs consist of different cations combined to chloride to extract BHC from water at 298 K.

| IL | AC-Water | AC-BHC | Selectivity BHC | Capacity BHC |
| --- | --- | --- | --- | --- |
| 1,2-dimethyl-3-ethyl-imidazolium Chloride | 21.19078 | 0.053917 | 393.0227 | 18.54687 |
| 1,3-dimethyl-imidazolium Chloride | 20.21586 | 0.018822 | 1074.064 | 53.12977 |
| 1-butyl-3-methyl-imidazolium Chloride | 26.55029 | 0.230305 | 115.2833 | 4.342074 |
| 1-butyronitrile-2,3-dimethylimidazolium Chloride | 11.97682 | 0.083641 | 143.1924 | 11.9558 |
| 1-butyronitrile-3-methylimidazolium Chloride | 12.20521 | 0.133921 | 91.13769 | 7.467111 |
| 1-ethyl-3-methyl-imidazolium Chloride | 23.65674 | 0.091803 | 257.6913 | 10.89294 |
| 1-methyl-3-propyl-imidazolium Chloride | 23.96017 | 0.12567 | 190.6597 | 7.95736 |
| 1-methyloxymethyl-3-methyl-imidazolium Chloride | 21.92992 | 0.120569 | 181.887 | 8.29401 |
| 2,3-dimethyl-1-hexyl-imidazolium Chloride | 28.63727 | 0.273878 | 104.5623 | 3.651267 |
| 2-hydroxyethyltrimethylammonium Chloride | 1.420579 | 0.011864 | 119.7362 | 84.2869 |
| Ethyl-dimethyl-propyl-ammonium Chloride | 21.5509 | 0.060975 | 353.4377 | 16.40013 |
| Methyl-tributyl-ammonium Chloride | 30.33179 | 0.263559 | 115.0855 | 3.794219 |
| Methyl-trioctyl-ammonium Chloride | 48.07506 | 0.308866 | 155.6502 | 3.237651 |
| tetra-N-butylammonium Chloride | 31.46732 | 0.209876 | 149.9329 | 4.764719 |
| Trimethyl-butyl-ammonium Chloride | 24.44623 | 0.12578 | 194.3564 | 7.950364 |
| 1-benzyl-3-methyl-pyridinium Chloride | 29.04543 | 0.300604 | 96.62355 | 3.326636 |
| 1-butyl-2-methyl-pyridinium Chloride | 24.49008 | 0.148831 | 164.5493 | 6.719018 |
| 1-butyl-3-methyl-pyridinium Chloride | 26.43289 | 0.236624 | 111.7084 | 4.226113 |
| 1-butyl-4-methyl-pyridinium Chloride | 26.66344 | 0.245654 | 108.5406 | 4.070763 |
| 1-ethyl-4-methyl-pyridinium Chloride | 22.92796 | 0.098416 | 232.9687 | 10.1609 |
| 1-propyl-4-methyl-pyridinium Chloride | 24.68027 | 0.166318 | 148.3919 | 6.01257 |
| Ethyl-pyridinium Chloride | 20.34607 | 0.026581 | 765.4489 | 37.62147 |
| 1-benzyl-1-methyl-pyrrolidinium Chloride | 26.89684 | 0.21952 | 122.526 | 4.555403 |
| 1-butyl-1-methyl-pyrrolidinium Chloride | 21.82854 | 0.082202 | 265.547 | 12.16513 |
| 1-octyl-1-methyl-pyrrolidinium Chloride | 30.72392 | 0.320619 | 95.82681 | 3.118965 |
| 1-pentyl-1-methyl-pyrrolodinium Chloride | 26.14541 | 0.208145 | 125.6118 | 4.804353 |
| 1-propyl-1-methyl-pyrrolidinium Chloride | 21.40043 | 0.065444 | 327.0031 | 15.28021 |
| tetra-N-butyl-phosphonium Chloride | 29.74638 | 0.172177 | 172.7668 | 5.807993 |
| tributyl-tetradecyl-phosphonium Chloride | 45.71586 | 0.191869 | 238.2662 | 5.211893 |
| trihexyl-tetradecyl-phosphonium Chloride | 56.17942 | 0.205061 | 273.9641 | 4.876591 |
| triisobutyl-methyl-phosphonium Chloride | 27.78461 | 0.192885 | 144.0473 | 5.184426 |
| methyl-trioctyl-phosphonium Chloride | 45.48112 | 0.213075 | 213.4515 | 4.693188 |
| Propyl-cholinium Chloride | 2.095116 | 0.205639 | 10.18833 | 4.862895 |

**Selectivity of ionic liquids**

a

**Figure S-1**: Calculated selectivity of various ILs towards a) Heptachlor b) Aldrin c) Gamma-Chlordane d) Endrin e) Methoxychlor at 298 K in water (Cation number refers to the identification key shown in **Table 2**).

**The capacity of ionic liquids**

**Figure S-2**: Calculated capacity of various ILs towards a) Heptachlor b) Aldrin c) Gamma-Chlordane d) Endrin e) Methoxychlor at 298 K in water (Cation number refers to the identification key shown in **Table 2**).

**Performance Index (P.I)**

a


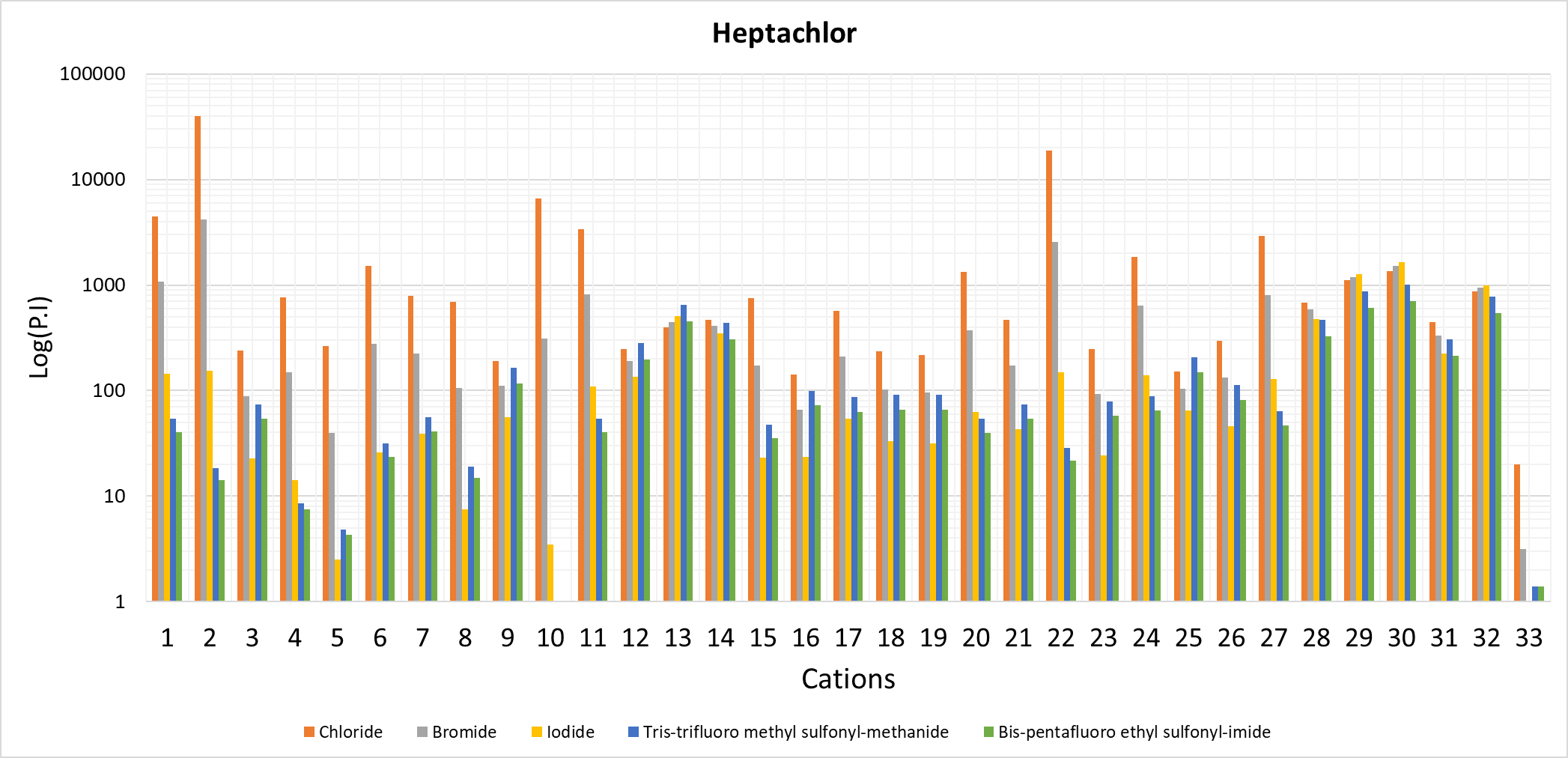


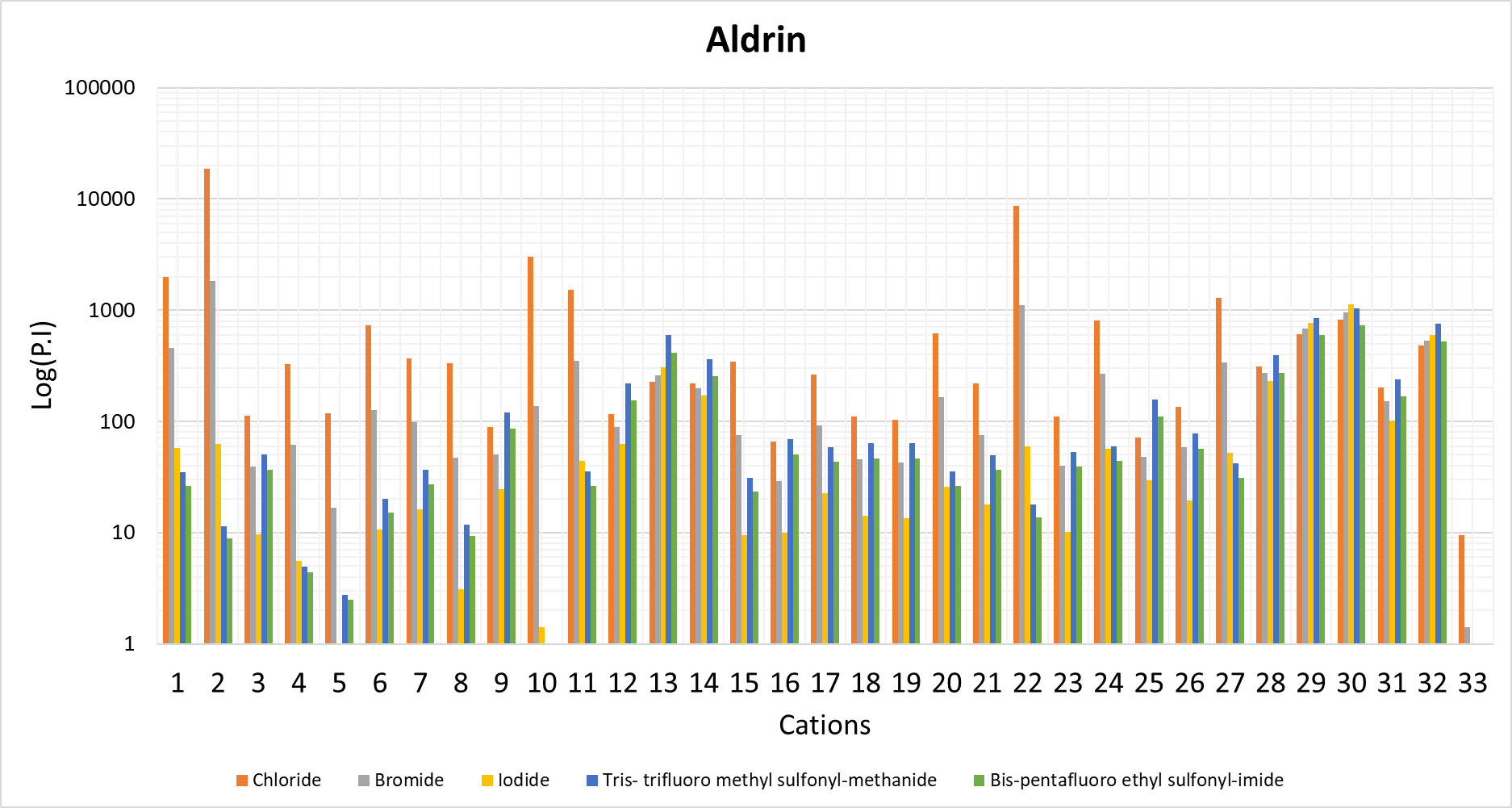


b


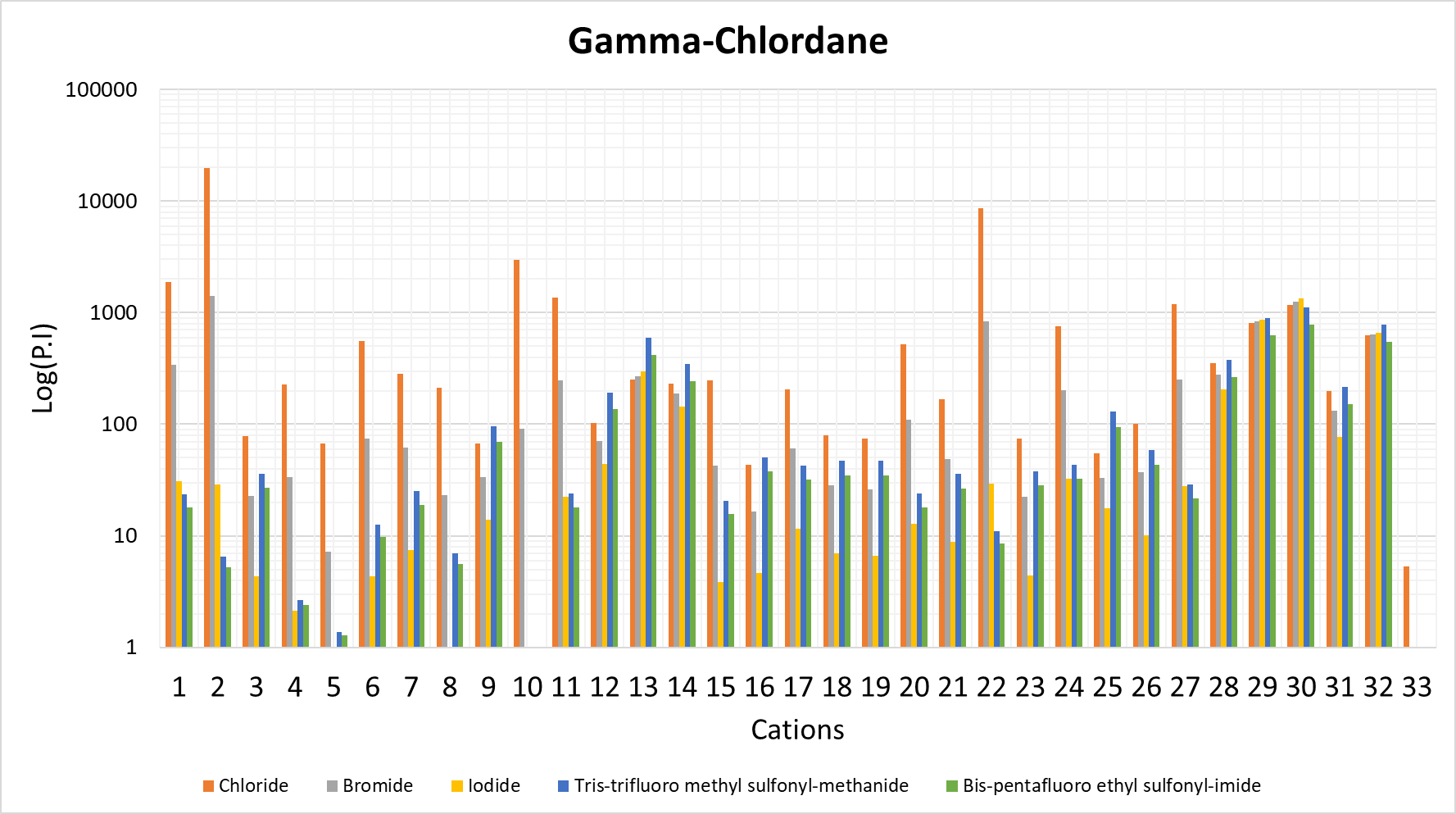


c


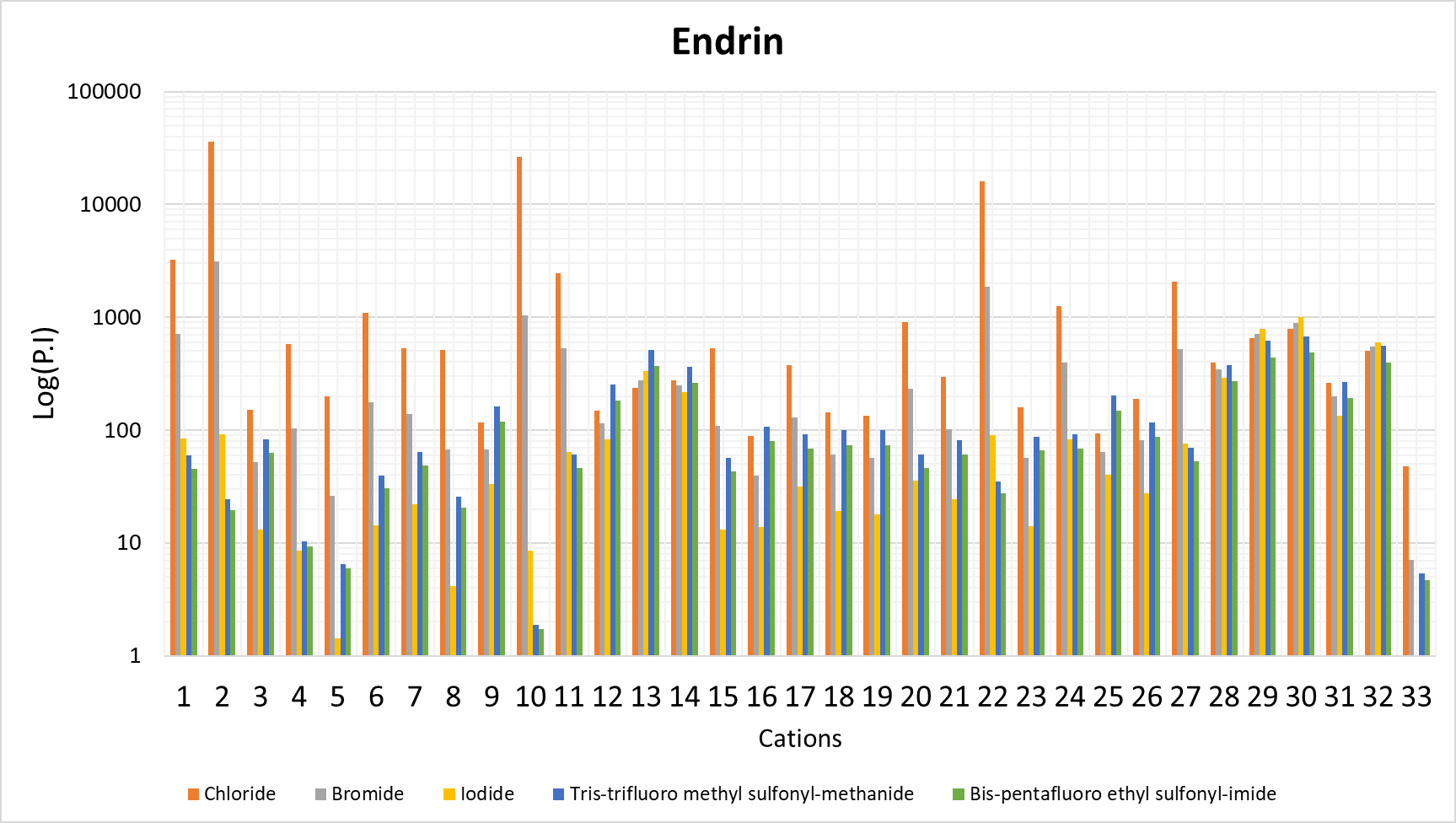


d


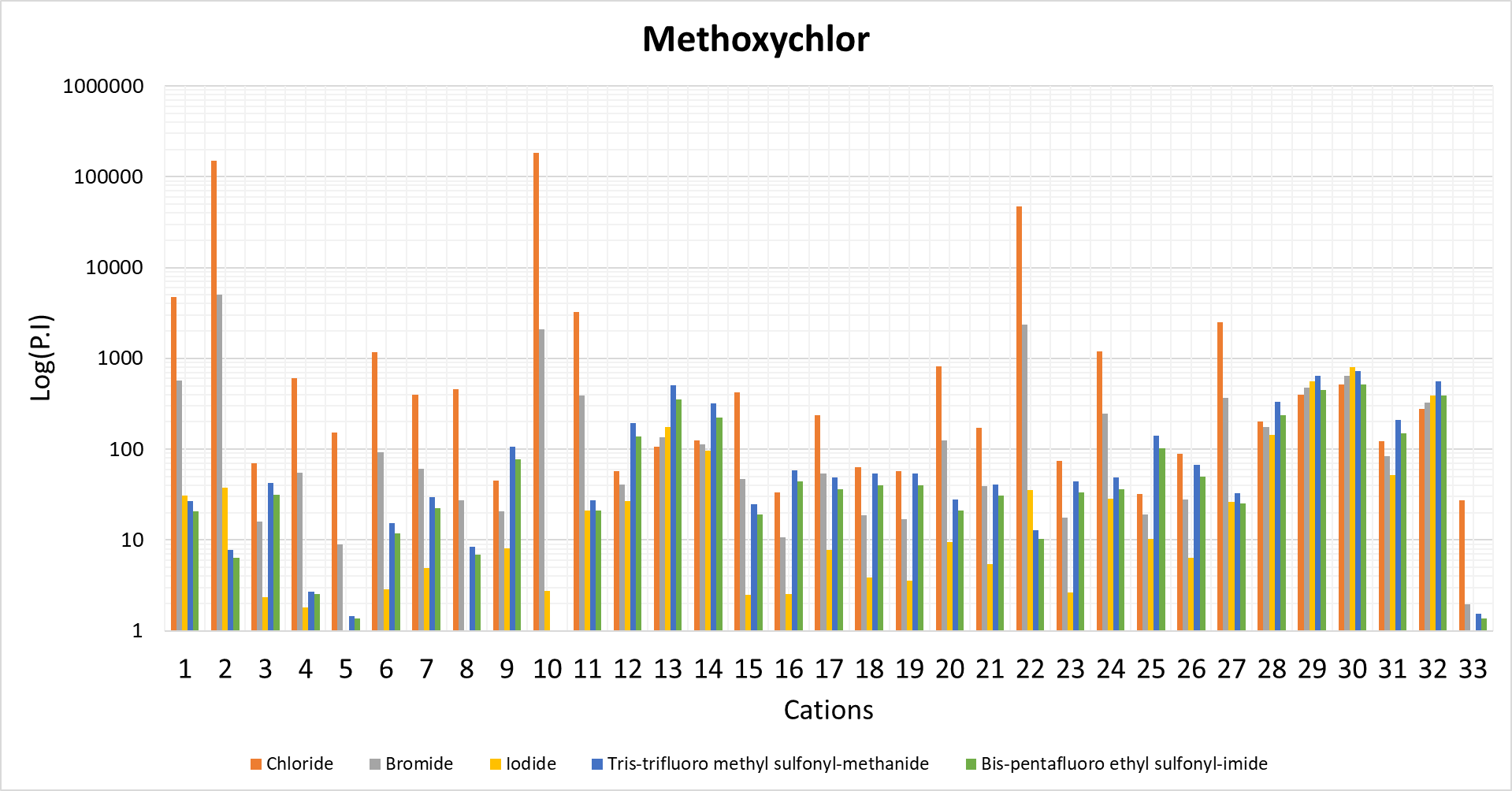


e

## Figure S-3: The performance index (P.I) of ILs for towards a) Heptachlor b) Aldrin c) Gamma-Chlordane d) Endrin e) Methoxychlor.
